# Computational Simulations to Guide Enzyme-Mediated Prodrug Activation

**DOI:** 10.3390/ijms21103621

**Published:** 2020-05-20

**Authors:** Milica Markovic, Shimon Ben-Shabat, Arik Dahan

**Affiliations:** Department of Clinical Pharmacology, School of Pharmacy, Faculty of Health Sciences, Ben-Gurion University of the Negev, 8410501 Beer-Sheva, Israel; milica@post.bgu.ac.il (M.M.); sbs@bgu.ac.il (S.B.-S.)

**Keywords:** prodrug, enzymatic activation, in silico modeling, DFT, quantum mechanics, molecular mechanics, molecular dynamics, molecular docking

## Abstract

Prodrugs are designed to improve pharmaceutical/biopharmaceutical characteristics, pharmacokinetic/pharmacodynamic properties, site-specificity, and more. A crucial step in successful prodrug is its activation, which releases the active parent drug, exerting a therapeutic effect. Prodrug activation can be based on oxidation/reduction processes, or through enzyme-mediated hydrolysis, from oxidoreductases (i.e., Cytochrome P450) to hydrolytic enzymes (i.e., carboxylesterase). This study provides an overview of the novel in silico methods for the optimization of enzyme-mediated prodrug activation. Computational methods simulating enzyme-substrate binding can be simpler like molecular docking, or more complex, such as quantum mechanics (QM), molecular mechanics (MM), and free energy perturbation (FEP) methods such as molecular dynamics (MD). Examples for MD simulations used for elucidating the mechanism of prodrug (losartan, paclitaxel derivatives) metabolism via CYP450 enzyme are presented, as well as an MD simulation for optimizing linker length in phospholipid-based prodrugs. Molecular docking investigating quinazolinone prodrugs as substrates for alkaline phosphatase is also presented, as well as QM and MD simulations used for optimal fit of different prodrugs within the human carboxylesterase 1 catalytical site. Overall, high quality computational simulations may show good agreement with experimental results, and should be used early in the prodrug development process.

## 1. Introduction

Prodrug is a drug derivative intended to undergo chemical/enzymatic activation and thereby release an active parent drug, which is then free to achieve its pharmacological effect in the body [1]. It is designed to improve stability, biopharmaceutical, pharmacokinetic and pharmacodynamic features of the parent drug [2]. The prodrug approach can enhance drug formulation strategy and accomplish improved administration; it can also be used in achieving site specificity, improving the therapeutic drug effect and drug safety [3,4]. Prodrugs constitute a particularly useful approach for altering physicochemical drug properties (e.g., solubility) and for optimizing drug-like features of active compounds (i.e., absorption, distribution, metabolism and excretion (ADME)) [5,6]. The prodrug approach has been proven efficacious for several therapeutic areas, including anticancer, anti-inflammatory, antiviral agents and angiotensin-converting enzyme inhibitors (ACEI) [7].

**Figure 1:**
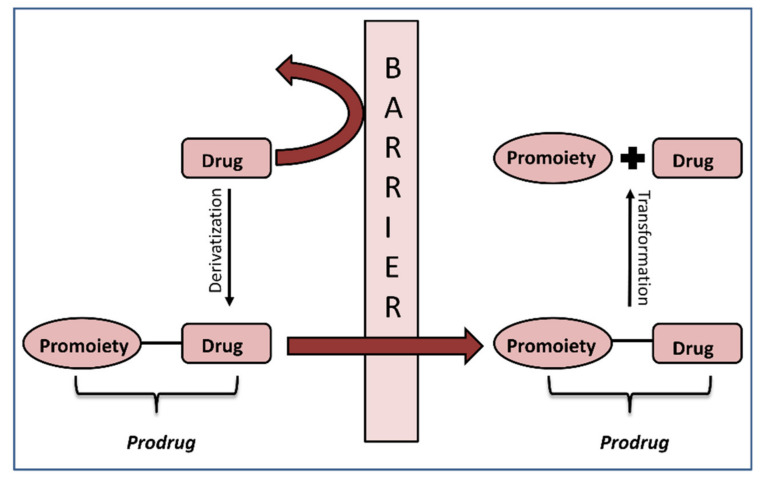
Illustration of the prodrug approach concept.

The crucial step in the successful prodrug design is the inclusion of the activation mechanism that releases an active parent drug from a prodrug molecule in an efficient/controlled way, in order to achieve a successful therapeutic effect. Prodrug activation can be achieved through enzyme-mediated hydrolysis or oxidation/reduction processes or through chemical degradation within the body, triggered via a particular stimulus. The main principle of the prodrug approach is illustrated in Figure 1. This work focuses on the enzyme-mediated prodrug activation and optimization of this process through in silico methods. Numerous enzymes can be involved in the activation of prodrugs; some examples include oxidoreductases like CYP450, and hydrolytic enzymes such as carboxylesterase, butyrylcholinesterase, acetylcholinesterase, paraoxonase, β-glucuronidase, matrix metalloproteinase, alkaline phosphatase (ALP), phospholipase A_2_ (PLA_2_), human valacyclovirase and others [8,9].

Prodrugs often contain ester/amide functional groups derived from the hydroxyl, carboxyl or amine group of the parent drug; ester/amide is then activated through hydrolysis or oxidation and the active drug moiety is liberated. Such prodrugs were often designed to enhance oral drug absorption [10]. The main obstacle with these molecules is the lack of ability to foresee the bioconversion of the prodrug to the active parent drug, and thus its therapeutic effect. This can be overcome by using novel computational modeling techniques, which can improve the prodrug design of drug molecules with hydroxyl/phenol/amine functional groups [11]. In addition, several in silico techniques are employed in the optimization of the prodrug structure or prodrug-enzyme complex, in order to facilitate enzymatic cleavage. They can be based on simple empirical methods (i.e., molecular docking), or rather complex methods (i.e., quantum/molecular mechanics or free energy perturbation). Computational simulations provide an important insight into the prodrug design and overall fitting of the prodrug to the enzyme catalytical site, therefore they should be conducted prior to experimental studies, in order to reduce cost and improve the efficiency of the prodrug development process. The following section provides a brief overview of the calculations and methods used for the optimization of the prodrug activation process.

## 2. In Silico Methods for Predicting Prodrug Activation

In silico methods that can predict binding affinity between an enzyme and a substrate can be simple, empirical methods, such as molecular docking, or more complex methods, based on the laws of physics, such as quantum mechanics/molecular mechanics (QM/MM) or free energy perturbation (FEP) [12,13]. Docking studies are a rapid, routine way for molecule selection and design, however their predicting precision is rather low; whereas QM/MM, as well as FEP methods are time/effort/money consuming, but are more reliable [14,15]. Docking studies have a lower level of accuracy due to limited molecule databases, poor docking position choice, unfitting target binding site etc. However, it is the most commonly used method in fast preliminary analysis in prodrug design. QM calculations, including ab initio, semi-empirical and density functional theory (DFT), as well as MM, are being gradually employed as tools to offer structure-energy calculations for the prediction of potential drugs/prodrugs [16]. Ab initio molecular orbital method is a QM method founded on Schrödinger equation with certain estimates; it is used in limited systems (not more than 30 atoms) due to costly computing time; it is a method to calculate the electronic distribution and other features of molecules [17,18]. Semi-empirical calculations, such as AM1, PM3, MINDO, MNDO, MINDO/3 and SAM1, are grounded on the Schrödinger equation, and they include certain features that fit the experimental results; these methods can be used for studying compounds with more than 50 atoms and thus are extensively utilized in QM. DFT is also a semi-empirical QM method that can provide structural/energy calculations for intermediate-size biological systems; in the last 30 years, it has been widely used in predicting and explaining the features of various materials [19]. MM is used for the computation of various physical properties (structure, energy, dipole moment) and is utilized in evaluating large biological systems such as proteins, but is restricted by numerous torsion angle computations in structurally different molecules. Free energy perturbation methods, such as molecular dynamics (MD), are a form of in silico simulation in which atoms/molecules are permitted to interact for a certain time period through estimates of known physics, giving an outlook of the motion of the atoms; nowadays, it is an extensively used method for drug/prodrug design [20]. MD is widely used method for gathering structural/functional information about various macromolecules. It can model proteins and biological macromolecules motion, usually using the laws of classical dynamics. MD simulations can be performed together with MM or with QM/MM techniques [21]. Some potential uses include the prediction of the Michaelis–Menten complex formation between the enzyme and the prodrug, entry of water molecule into the active site of a particular enzyme, and evaluation of the steric hindrance between the prodrug and the enzyme [20]. All these computational approaches may be used for an intelligent design of novel prodrugs.

## 3. Computational Optimization of Enzyme-Mediated Prodrug Activation

Prodrug activation can be mediated through different enzymes, from oxidoreductases like Cytochrome P450 to hydrolytic enzymes such as carboxylesterase, phospholipase A_2_, β-glucuronidase, alkaline phosphatase and others. Numerous enzymes exhibit significant conformational modifications during the reaction cycle, and the function/relationship of such modifications to the chemical reaction steps should be investigated. In addition, enzymes have complex dynamics, with a range of internal motions, often including ones crucial to their function. This section provides an overview of particular enzyme-mediated prodrug activation and various in silico modeling techniques used for the optimization of the activation process.

### 3.1. Cytochrome P450 (CYP450)

There is substantial proof that the genetic polymorphisms of Cytochrome P450 (CYP450) enzymes contribute to the inconsistency in the activation of various prodrugs and hence to the effectiveness/safety of the drugs undergoing this bioactivation pathway [22]. The CYP450 enzymes mainly involved in the drug metabolism are CYP1A2, 2B6, 2C8, 2C9, 2C19, 2D6, 2E1, 3A4 and 3A5, which makes them of key interest for the activation of the prodrugs as well [23]. CYP3A4 is present in the hepatic tissues in the highest concentration, and is accountable for the oxidation of nearly two-thirds of all known drugs; nevertheless, other P450 are significant for drug metabolism and prodrug activation as well [24], and their expression is different in various tissues; therefore, they can be used to selectively target prodrugs to particular tissues.

CYP2C9 is responsible for the metabolism of approximately 15% of drugs on the market. Some variants of CYP2C9 show decreased enzymatic activity due to polymorphism, which can affect the clinical response of certain drugs. The uncommon allelic variant, CYP2C9.30 (which demonstrates < 50% activity when compared to wild type enzymes) is found in Japanese people and is linked to a decreased therapeutic response of the prodrug losartan [25]. MD simulations alongside energetic analyses were shown to be successful in elucidating the mechanism of amino acid (AA) replacement that influences drug metabolism/regioselectivity of CYP450 [26]. The CYP2C9 and wildtype/A477T models were solvated with TIP3P water molecules, and systems with ~60,000 atoms were used. The simulations included CHARMM36 force field, and for losartan parameters, the DFT QM calculations were used. Structures of the substrate binding pocket, substrate docking, and channel dynamics were also determined using suitable software. The mutant enzyme showed higher rigidity of enzyme fragments involved in the recognition of the substrate, which could in turn affect the entrance and fitting of the substrate. MD simulations demonstrated that the A477T mutation present in CYP2C9.30 variant caused bigger rigidity of the key substrate recognition sites (SRS1 and SRS5) and the shifting of the β turn 4 of SRS6 toward helix F, thus decreasing the substrate access to some protein channels. The behavior of substrates, like losartan, was observed, and it was seen that the A477T mutant can modify the metabolism of some substrates, when compared to wildtype. The dynamics of substrate binding showed changes in substrate channel access and fitting to the active site, which could be responsible for altered catalytic enzymatic activity. Identified conformations of enzyme and substrates could be used for predicting suitable substrates of drug-drug interactions [27].

**Figure 2:**
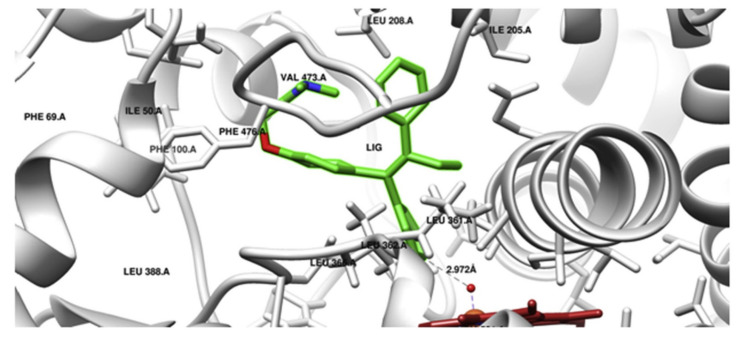
Molecular docking (Autodock) of tamoxifen with CYP2C9 (PDB ID 1OG5). Tamoxifen (LIG) in 4-hydroxy orientation is presented in green, and heme is shown in red. The prodrug 4-hydroxylation site is towards oxyferryl species (5 Å distance); the Ligplot analysis non-bonded contacts of tamoxifen with amino acid residues of SRS5 (LEU 362, LEU 366), SRS6 (PHE 476, ALA 477), SRS2 (ILE 205, LEU208), SRS4 (ALA 297, THR 301) were observed. Docking of tamoxifen with CYP2C9 is consistent with the tamoxifen metabolism pathway. Reproduced with permission from [28].

A similar scenario was shown for prodrug tamoxifen, which is transformed via CYP2C9 to a more potent metabolite 4-hydroxytamoxifen. In order to determine the effect of CYP2C9 genetic polymorphism on the phenotypic drug response, interactions of AA substitutions in CYP2C9 variants (CYP2C9 R144C (*2), I359 L (*3), D360E (*5), R150H (*8), R335W (*11) and L90 P (*13)) in the company of tamoxifen were studied using the computational structural biology approach. Tamoxifen docking conformation, done with the crystal structure of the wild-type enzyme form, showed the formation of 4-hydroxytamoxifen by CYP2C9 (Figure 2). MD simulations of CYP2C9 variants with AA substitutions *2,*3,*5,*8,*11 and *13 connected with tamoxifen did indeed show structural changes. Changes were shown in the substrate specificity determining region and the substrate access or leaving channels. Therefore, the structural variations in CYP2C9*2, *3, *5, *8, *11 and *13 CYP2C9 variants are responsible for the variable enzyme-mediated activation of tamoxifen [28].

Paclitaxel has limited solubility, which restricts its widespread use. Many paclitaxel prodrugs were designed in order to improve its aqueous solubility. A recent study presents a quantitative structure property relationship (QSPR) model, which could serve as a basis for novel paclitaxel prodrug design with improved solubility. Molecular docking and molecular dynamics simulation were then used for the metabolic study with CYP1A2 enzyme, which revealed that the substituent groups in the prodrug could indeed be metabolized by the CYP1A2 enzyme [29].

### 3.2. Carboxylesterase

Prodrugs with ester bonds are frequently hydrolyzed via human carboxylesterase (hCE). Two major hCE enzyme classes are present in humans, hCE1 and hCE2. hCE1 has smaller catalytic site, and thereby cleaves smaller substrates, whereas hCE2 is reported to have a bigger catalytic domain [30]. However, both forms of the enzyme contain a catalytical site that is characterized by serine, histidine and glutamine; hydroxyl group in the serine can attack the carbonyl group of the ester prodrug, and the remaining 2 AA stabilize the complex. These molecular targets were employed in studying novel prodrugs of epalrestat and natural antioxidant products for diabetes complications [31]. MD and QM techniques were used in the process of prodrug design leading to the optimal accommodation within the catalytical site of hCE1. Most of the given prodrugs are monocyclic phenols, thus, due to the size of its catalytical site, the hCE1 enzyme was selected for analysis.

**Figure 3:**
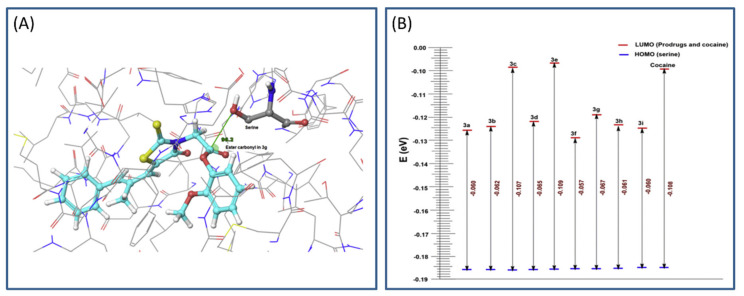
Representation of highest occupied molecular orbital-lowest unoccupied molecular orbital (HOMO-LUMO) gap (**A**) and Burgi–Dunitz angle between hydroxyl group of serine and ester carbonyl of the optimal prodrug (epalrestat-guaiacol) (**B**). Reproduced with permission from [31].

The main process in the hCE1 catalytical activation is the nucleophilic attack of serine hydroxyl group on the carbonyl ester group of the prodrug (based on frontier molecular orbital approach). Calculations including geometrical parameters (Burgi–Dunitz angle governing the nucleophilic attack and distance) and QM factors, such as highest occupied molecular orbital-lowest unoccupied molecular orbital (HOMO-LUMO) energy gap, were performed to determine the prospect of hydrolytic cleavage of the ester prodrugs via esterase (Figure 3A). HOMO-LUMO difference can provide an insight into the mechanism of transfer of electrons from protein to the prodrug within the catalytic domain. The HOMO and LUMO calculations were performed using Schrödinger software, and DFT analyses (with local density approximation functional) were engaged to calculate the HOMO-LUMO energy for all structures. A gap in the energy difference within the HOMO and LUMO can be a certain stability index; high gap means high stability (Figure 3B), and low chemical reactivity, and vice versa [32]. The MD simulation of designed prodrugs showed that the monocyclic prodrugs are more suitable substrate for hCE than bicyclic antioxidants, which was confirmed via QM as well. Governed by in silico predictions, prodrugs were synthesized and evaluated in the in vivo system; bearing in mind the in silico predictions, the most promising prodrug was evaluated for its in vivo antioxidant activity [31].

### 3.3. Phospholipase A_2_ (PLA_2_)

**Figure 4:**
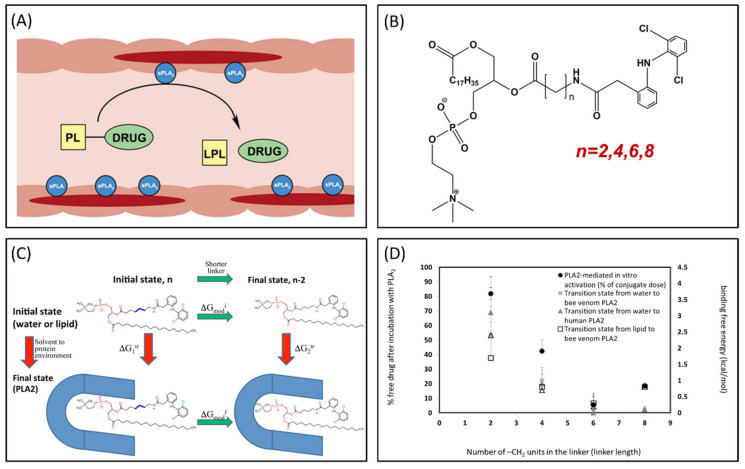
(**A**) Illustration of phospholipid (PL)-prodrug activation in phospholipase A_2_ (PLA_2_)-rich inflamed intestinal tissues of Inflammatory Bowel Disease (IBD) patients; (**B**) Structure of PL-diclofenac prodrug; (**C**) Thermodynamic cycle used for relative binding free energy calculations of PL-diclofenac prodrugs in the transition state complex of PLA_2_; and (**D**) In vitro/in silico correlation: binding free energies in PLA_2_transition state (kcal/mol) vs. in vitro activation for different PL-diclofenac prodrugs (% of intact complex). Reproduced with permission from [37,40].

Phospholipase A_2_ (PLA_2_) is overexpressed in many inflammatory conditions and cancer [33,34,35]. PLA_2_ is an enzyme responsible for hydrolyzing the *sn*-2 bond of the phospholipid (PL) [36]. Our group studies PL-prodrugs consisting of the drug covalently bound to the *sn*-2 position of the PL. We aim to exploit the PLA_2_ enzyme in order to hydrolyze the *sn*-2 bond of the prodrug, thereby releasing the drug specifically at the site of action, where the enzyme PLA_2_ is overexpressed (Figure 4A) [37]. Direct conjugation between the PL and the drug demonstrated lack of activation by PLA_2_ [38], therefore, our PL-prodrugs contain different linker lengths between the PL backbone and the drug in the *sn-*2 position of the PL; different linker length resulted in different rate and extent of activation [8]. We developed an MD simulation in order to determine energetic changes of the PL-drug conjugates in the active site of PLA_2_ [39,40,41,42]. An example of the PL-diclofenac prodrug structure is presented in Figure 4B. PLA_2_-mediated activation will occur only if the prodrug takes on a well-defined transition state geometry of the enzyme active site, characterized by interactions of the *sn*-2 carbonyl oxygen with the calcium atom from the PLA_2_ active site, together with the protein His residue that activates a water molecule, used for the nucleophilic attack on the acyl group of the prodrug. The PL-drug concentration in the PLA_2_ transition state geometry and kinetics of the cleavage are evaluated by the binding free energy of the prodrug within the enzyme active site. Lower binding free energy correlates with a higher degree of binding between the PL-drug conjugate and the enzyme. The differences in the transition state binding energy in the PLA_2_ was calculated through decreasing/increasing the linker length. We used the thermodynamic integration (TI) method to compute the free energy of eliminating the -CH_2_ units from the linker (Figure 4C). TI calculates the free energy difference among the two states by ensemble-averaging the enthalpy variation along the path that connects the two states. The free energy of attaching the drug to the shorter linker was calculated using the umbrella sampling (US) and weighted histogram analysis methods (WHAM). Prodrugs containing diclofenac [43], indomethacin [44,45,46], and methotrexate [42] were evaluated through this simulation [39,40,42]. Optimal linker length was evaluated, and confirmed through in vitro [43,44] and in vivo [44] studies, with good correlation to in silico studies (Figure 4D).

Furthermore, MD and DFT simulations were performed on thio-ester pro anticancer ether lipid (S-ProAEL) and the mechanisms behind the PLA_2_-mediated activation were explained. It was shown that the presence of the water molecule has a significant part in the activation of different derivatives of PL-prodrugs (as well as for thio-esters). Further studies showed the distinction between the hydrolysis rate of the S-ester vs. the natural O-ester; significantly different ester conformation and a longer space between the carbonyl and the water molecule in the S-ester when compared to O-ester [47].

### 3.4. Cholinesterase

In a recent study, novel prodrugs for Alzheimer’s disease targeting 2 specific targets, dual-specificity tyrosine phosphorylation-regulated kinase 1A (DYRK1A) and cholinesterase (ChE), were designed and synthesized [48]. The ChE and DYRK1A inhibitors were connected through a carbonate link. It was shown that a prodrug is inactive against both DYRK1A and ChEs and exhibits potent inhibition of ChEs only, through the oxidized form. The evaluation of promising binding interaction that contributed to the inhibition of ChE was performed via molecular docking (using Autodock Vina software). Supporting the likely interaction of prodrug with the peripheral anionic site of the enzyme, molecular docking suggests that the benzothiazole can be π-stacked with the tryptophan 286 residue of the peripheral anionic site, with a possible π-sulfur interaction among the thiazole moiety of the ligand and the same residue. The inhibition of ChE by compound of the prodrug could be clarified via a pseudo-irreversible mechanism. The carbonate link installed between the two inhibitors permitted transient masking of the inhibitory activity of the drug inhibitor against DYRK1A [48].

### 3.5. Alkaline Phosphatase

In an attempt to concentrate radioactive drugs inside the solid tumors, a novel technology is utilized: enzyme-mediated cancer imaging and therapy. A radioactive quinazolinone prodrug, ammonium 2-(2′-phosphoryloxyphenyl)-6-[^125^I]iodo-4-(3H)-quinazolinone (^125^IQ_2-P_) is water-soluble, and can be hydrolyzed by alkaline phosphatase to a water-insoluble, 2-(2′-hydroxyphenyl)-6-[^125^I]iodo-4-(3H)-quinazolinone (^125^IQ_2-OH_). Upon enzymatic activation, the water-soluble molecule liberates its prosthetic group and the consequential molecule is water-insoluble and precipitates; this allows it to be permanently trapped within the extracellular spaces of targeted solid tumors. Two isoforms of the prodrug are present (IQ_2-P(I)_ and IQ_2-P_), and their structural alterations were studied via molecular modeling and docking methods, in order to elucidate the interaction among the prodrugs and the enzyme human placental alkaline phosphatase (PLAP), a membrane-bound hydrolytic enzyme of the tumor cell. The PLAP active site was well-defined using the AutoGrid module, and metal ions were modeled by Amber force field potentials (AutoDock). IQ_2-P(I)_ and IQ_2-P_ were docked into PLAP, and both binding free energy (∆G_binding_) and inhibition constant (K_i_) were calculated. Molecular docking results, following energy minimization and geometric optimization (until there was no resistance among ligand, metal ion, water molecules, and PLAP), revealed that only IQ_2-P_ accommodated the PLAP active binding site and interacts with the catalytic AA Ser92 that plays an important role in the hydrolytic process. The fact that IQ_2-P_ is a more suitable substrate was shown by the binding free energy (∆G_binding_) of the isoforms to PLAP; the binding affinity of IQ_2-P_ was ~170 fold stronger than that of IQ_2-P(I)_. This was also confirmed through in vitro/in vivo studies [49].

### 3.6. Human Valacyclovirase

Valacyclovir prodrug was developed to overcome the low permeability of acyclovir. It was discovered that the conversion of valacyclovir to acyclovir occurs through enzyme valacyclovirase (VACVase), and that this enzyme presents an important therapeutic target for prodrug design [50]. For instance, VACVase is a suitable target for AA ester prodrugs; it is also known to hydrolytically activate antiviral nucleoside prodrugs, valganciclovir, producing AA, L-valine and corresponding parent drugs ganciclovir [51].

**Figure 5:**
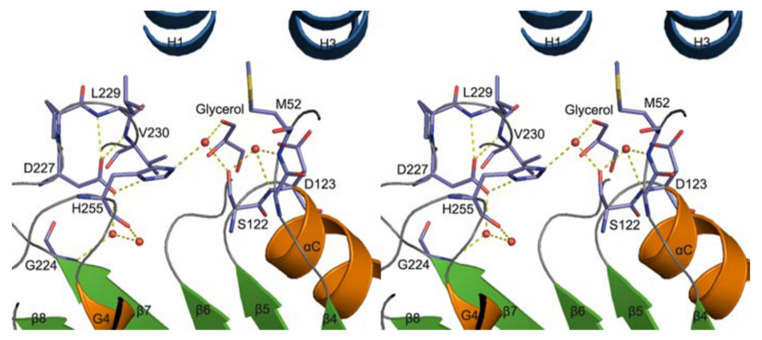
Valacyclovirase active site stereoview (red circles are water molecules). Reproduced with permission from [53].

A homology model of VACVase was developed (via molecular operating environment software) to investigate the substrate specificity of different nucleoside analogues which offer a deeper understanding of catalytic and active site residues [52]. The docking of substrates and VACVase was performed with the MMFF94 force field with the solvation term by MOE-DOCK. It was shown that the model contains residues S122, H255 and D227 close to the catalytic triad, and charge-charge interaction sites, M52 or D123 for the R-amino group. Structural affinity of VACVase for hydrophobic amino acyl groups and restricted preference for the secondary alcohol substrates was shown. The reason for this might be attributable to the hydrophobic acyl-binding site consisting of I158, G161, I162, and L229 residues and the spatial limitation surrounding the active site by a loop from one side, serine and histidine on the other side, and L53 and L179 on the top. An additional study showed that VACVase has a specific binding mode and affinity for amino acid esters [53]; it contains a distinctive active site characterized by hydrophobic acyl pocket, a confined negative electrostatic potential, large open leaving group accommodating groove, and AA residue, Asp-123 (Figure 5) [53] This is the first instance that a residue following nucleophile has a side chain heading for the substrate binding pocket and has an important part in substrate discrimination in serine hydrolases [53]. This study concluded that the enzyme functions as a specific α-amino acid ester hydrolase. The structure activity profile of the VACVase model is a useful tool for the future design of nucleoside prodrugs [54,55].

Another study showed that the rate of acyclovir prodrug hydrolysis could be evaluated based on the structural properties of the linker used [56]. It was shown that the rate-limiting step may be the creation of tetrahedral intermediate depending on the substitution choice. This insight could allow development of less hydrophilic prodrug system and controlled parent drug release. MM methods such as ab initio and DFT could provide better understanding of intramolecular processes, with the objective to elucidate the underlying parameters that influence the rate-determining step in prodrug activation. These methods have a crucial role in leading the intelligent design of novel prodrugs.

## 4. Discussion

Advancement in computing power and algorithms allows for the rapid development and innovation of the in silico tools that may advance our understanding of the biological systems. Molecular docking is a valuable tool for predicting binding modes for ligands, in particular protein targets, and can be used for target-based design of the prodrug (e.g., Section 3.4 and Section 3.6); it is simple, relatively fast and has relatively low reliability [57]. Nevertheless, it cannot recognize relevant binding modes inside large and flexible binding sites, in cases when the enzyme dynamics should not be disregarded. This is where classical MD simulations, as well as accelerated MD and metadynamics, can help, accounting for the atomic descriptions of enzyme and prodrug dynamics, as well as the free energy of the entire conformational ensembles. These methods offer both structural and dynamic features of the enzyme (in a ≤ milisecond), which could predict likely binding sites, and propose the optimal enzyme-prodrug complex. The MD simulations are widely used and have short simulation times, however the absence of optimized tools and representation standards remains an obstacle. MM computation is focused on structures, energy, dipole moment and other physical properties, and does not take into account chemical reaction. QM methods calculate the distribution of electrons in molecules using the laws of quantum mechanics; they are extremely important in order to understand what happens in the chemical reaction (the change in the electron distribution in a reaction when bonds are broken and/or formed). For instance, nuclear tunneling is very significant in enzyme reactions that involve hydrogen transfer [58]. Nonetheless, it includes a relatively small number of atoms, which limits the understanding of the whole biological systems [59]. A potent way of overcoming these limitations is the use of QM/MM calculations, in which the system is divided into QM and MM regions, where QM regions deal with the electrons in a small area (i.e., the active site of an enzyme), and a larger part of the protein, the solvent and any other groups connected to the protein are modeled by MM. In order to include the environmental effects on the enzyme reaction, the QM and MM regions ought to interact, meaning that the QM region should be under the influence of the MM atoms. For instance, this method was shown to be successful in elucidating the catalytic function of active-site residues such as conserved proline in flavin-dependent monooxygenases [60]. It is sometimes said the QM/MM are hybrid simulations, in the sense that they are a combination of methods [61]. MM methods are also used in MD simulations and docking. Altogether, through the combination of several modeling methods, better prediction of enzyme-prodrug interaction may be obtained than the use of one technique alone. An overview and limitations of the key computational approaches used in the enzyme-mediated prodrug activation are presented in Table 1.

Numerous prodrugs have been designed and developed aiming to improve the effectiveness of certain drugs and to overcome pharmaceutical/pharmacokinetic obstructions that occur upon drug administration (e.g., low stability, poor absorption, low solubility, reduced patient compliance) [5,66]. Many endogenous enzymes can be used for prodrug activation. In addition, recent advancement in the delivering of prodrugs to the specific tissue/organ employed non-endogenous enzymes for the site-specific activation of prodrugs: directed enzyme prodrug therapy (DEPT) [67]. However, prodrug development process in both cases is costly in terms of time, money and effort. The above mentioned in silico methods may provide trustworthy predictions, reducing the time and experimentation attempts, and making the overall process more affordable.

One of the main uses of improving the prodrug activation process is the optimization of the prodrug structure. Alongside PL-diclofenac, indomethacin and methotrexate, the use of different linkers/spacers/side chains was studied through different in silico methods; some examples include acyclovir prodrugs [56], PL-retinoid prodrug [68], anticancer ether lipids [47,69] and atovaquone [70]. The use of such groups allows the liberation of the parent drug at different, adjustable rates, depending upon the structural properties of the group itself. This approach, alongside predicting the molecular target (enzyme) for the prodrug, highlights the importance of the modern computational approached in the prodrug design. These computational methods reduce the need for numerous chemical synthesis and a long prodrug development process. In many cases, there was high correlation with experimental results (i.e., Figure 4D) [40,47,71]. A mechanism of prodrug intramolecular processes could also be used for the design of novel prodrug linkers. MM, DFT and ab initio methods were used, aiming to allocate the parameters that are influencing the rate-determining step and leading the process rate [16,70].

The molecular revolution has changed the process of prodrug development significantly. While the traditional prodrug approach focused on physicochemical drug features (e.g., charge, lipophilicity), the modern prodrug approach takes into account the molecular and cellular parameters, such as protein (e.g., enzymes, transporters) expression and distribution. Reducing the empirical features by using the targeted prodrug approach, while optimizing the activation process through in silico studies, endorses an effective overall process, as the outcomes may be considerably more predictable. Additionally, future progress should be made in the field of organic chemistry reactions that may be used in the development of certain prodrugs. Excellent understanding of the intramolecular reactions of prodrugs is essential for the advancement of this field as well. Since molecular docking, computational simulations and physical methods have their own limitations, a combination of physical methods and computational simulations yields more precise, biologically relevant results [72]. For instance, molecular docking and MD simulations come across some difficulties when it comes to predicting transient states in the enzyme reactions; the combination with QM methods (such as DFT) can help to overcome these limitations. In the coming years, an even wider use of computational approach is expected; different molecular orbital methods such as DFT and ab initio, as well as MM and MD simulations, provide a steppingstone for modern prodrug design.

## 5. Conclusions

The recent development of computational power allows powerful in silico methods to be used in the evaluation of the enzyme activity, catalysis and substrate specificity. Excellent knowledge of enzyme structure can help in evaluating enzyme-mediated prodrug activation, by offering new information regarding the structural requirements of prodrugs in order to accomplish optimal enzyme activation. QM, MM and MD simulations may provide an exceptionally thorough analysis of the main enzyme barriers, and understanding of the parameters needed for successful prodrug discovery/development. Prospective modeling studies should be performed prior to the prodrug synthesis and in vitro evaluation, in order to decrease the number of experimentations and save resources. Combined approaches, including novel in silico simulations of prodrug/enzyme complex, with rational prodrug design, present an important direction for the future development of the prodrug approach.

## Figures and Tables

**Table 1:** Comparison of computational methods used in enzyme/prodrug simulations.

	Molecular Docking	Quantum Mechanics	Molecular Mechanics	Free Energy Perturbation
**Purpose**	Predicting ligand-target interactions at a molecular level [62]	Study reactions in molecular systems [63]	Protein structure and dynamics (not applicable to chemical reactions) [59]	Computing free energy differences between an enzyme and a substrate [64]
**Calculation**	Algorithms	Schrödinger equation	Empirical methods	Laws of classical dynamics
**Applications**	Various systems	Small system size (e.g., isolated active sites)	Large systems (proteins, large crystal structures and relatively large solvated systems)	Proteins and other biological macromolecules
**Limitations**	Low reliability, cannot be used when protein dynamics should not be ignored [62]	Difficulty in understanding large biological system (used for up to a few hundred atoms); high computational time and resource [63]	Large number of unique torsion angles present in different molecules [65]	High computational costs; absence of optimized tools and representation standards [20]

## References

[1] Rautio J., Kumpulainen H., Heimbach T., Oliyai R., Oh D., Jarvinen T., Savolainen J. (2008). Prodrugs: Design and clinical applications. Nat. Rev. Drug Discov..

[2] Testa B. (2009). Prodrugs: Bridging pharmacodynamic/pharmacokinetic gaps. Curr. Opin. Chem. Biol..

[3] Dahan A., Zimmermann E.M., Ben-Shabat S. (2014). Modern prodrug design for targeted oral drug delivery. Molecules.

[4] Stella V.J. (2010). Prodrugs: Some thoughts and current issues. J. Pharm. Sci..

[5] Stella V.J., Nti-Addae K.W. (2007). Prodrug strategies to overcome poor water solubility. Adv. Drug Deliv. Rev..

[6] Testa B. (2004). Prodrug research: Futile or fertile?. Biochem. Pharmacol..

[7] Huttunen K.M., Raunio H., Rautio J. (2011). Prodrugs—From serendipity to rational design. Pharmacol. Rev..

[8] Dahan A., Markovic M., Aponick A., Zimmermann E.M., Ben-Shabat S. (2019). The prospects of lipidic prodrugs: an old approach with an emerging future. Future Med. Chem..

[9] Yang Y.-H., Aloysius H., Inoyama D., Chen Y., Hu L.-Q. (2011). Enzyme-mediated hydrolytic activation of prodrugs. Acta Pharm. Sin. B.

[10] Rautio J., Meanwell N.A., Di L., Hageman M.J. (2018). The expanding role of prodrugs in contemporary drug design and development. Nat. Rev. Drug Discov..

[11] Dahan A., Khamis M., Agbaria R., Karaman R. (2012). Targeted prodrugs in oral drug delivery: The modern molecular biopharmaceutical approach. Expert Opin. Drug Deliv..

[12] Kapetanovic I.M. (2008). Computer-aided drug discovery and development (CADDD): In silico-chemico-biological approach. Chem. Biol. Interact.

[13] Lesyng B., McCammon J.A. (1993). Molecular modeling methods. Basic techniques and challenging problems. Pharmacol. Ther..

[14] Hernandez B., Luque F.J., Orozco M. (2000). Mixed QM/MM molecular electrostatic potentials. J. Comput. Aided Mol. Des..

[15] Keinan S., Frush E.H., Shipman W.J. (2018). Leveraging cloud computing for in-silico drug design using the quantum molecular design (QMD) framework. Comput. Sci. Eng..

[16] Karaman R., Fattash B., Qtait A. (2013). The future of prodrugs—Design by quantum mechanics methods. Expert Opin. Drug Deliv..

[17] Chen M., Ko H.Y., Remsing R.C., Calegari Andrade M.F., Santra B., Sun Z., Selloni A., Car R., Klein M.L., Perdew J.P. (2017). Ab initio theory and modeling of water. Proc. Natl. Acad. Sci. USA.

[18] Kamerlin S.C., Haranczyk M., Warshel A. (2009). Progress in ab initio QM/MM free-energy simulations of electrostatic energies in proteins: Accelerated QM/MM studies of pKa, redox reactions and solvation free energies. J. Phys. Chem. B.

[19] Yeston J. (2017). Whither the density in DFT calculations?. Science.

[20] Hospital A., Goñi J.R., Orozco M., Gelpí J.L. (2015). Molecular dynamics simulations: Advances and applications. Adv. Appl. Bioinform. Chem..

[21] Field M.J. (2002). Simulating enzyme reactions: Challenges and perspectives. J. Comput. Chem..

[22] Testa B., Kramer S.D. (2007). The biochemistry of drug metabolism—An introduction: Part 2. Redox reactions and their enzymes. Chem. Biodivers.

[23] Ortiz de Montellano P.R. (2013). Cytochrome P450-activated prodrugs. Future Med. Chem..

[24] Huttunen K.M., Mahonen N., Raunio H., Rautio J. (2008). Cytochrome P450-activated prodrugs: Targeted drug delivery. Curr. Med. Chem..

[25] Yin T., Maekawa K., Kamide K., Saito Y., Hanada H., Miyashita K., Kokubo Y., Akaiwa Y., Otsubo R., Nagatsuka K. (2008). Genetic variations of CYP2C9 in 724 Japanese individuals and their impact on the antihypertensive effects of losartan. Hypertens. Res..

[26] Dodani S.C., Kiss G., Cahn J.K.B., Su Y., Pande V.S., Arnold F.H. (2016). Discovery of a regioselectivity switch in nitrating P450s guided by molecular dynamics simulations and Markov models. Nat. Chem..

[27] Louet M., Labbé C.M., Fagnen C., Aono C.M., Homem-de-Mello P., Villoutreix B.O., Miteva M.A. (2018). Insights into molecular mechanisms of drug metabolism dysfunction of human CYP2C9*30. PLoS ONE.

[28] Manish M., Lynn A.M., Mishra S. (2020). Cytochrome P450 2C9 polymorphism: Effect of amino acid substitutions on protein flexibility in the presence of tamoxifen. Comput. Biol. Chem..

[29] Merali Z., Ross S., Pare G. (2014). The pharmacogenetics of carboxylesterases: CES1 and CES2 genetic variants and their clinical effect. Drug Metab. Drug Interact..

[30] Munjal N.S., Shukla R., Singh T.R. (2019). Chemometric approach to estimate kinetic properties of paclitaxel prodrugs and their substructures for solubility prediction through molecular modelling and simulation studies. J. Chemom..

[31] Vyas B., Choudhary S., Singh P.K., Singh A., Singh M., Verma H., Singh H., Bahadur R., Singh B., Silakari O. (2018). Molecular dynamics/quantum mechanics guided designing of natural products based prodrugs of Epalrestat. J. Mol. Struct..

[32] Fukui K., Yonezawa T., Shingu H. (1952). A molecular orbital theory of reactivity in aromatic hydrocarbons. J. Chem. Phys..

[33] Minami T., Shinomura Y., Miyagawa J., Tojo H., Okamoto M., Matsuzawa Y. (1997). Immunohistochemical localization of group II phospholipase A2 in colonic mucosa of patients with inflammatory bowel disease. Am. J. Gastroenterol..

[34] Minami T., Tojo H., Shinomura Y., Matsuzawa Y., Okamoto M. (1994). Increased group II phospholipase A2 in colonic mucosa of patients with Crohn’s disease and ulcerative colitis. Gut.

[35] Yarla N.S., Bishayee A., Vadlakonda L., Chintala R., Duddukuri G.R., Reddanna P., Dowluru K.S. (2016). Phospholipase A2 isoforms as novel targets for prevention and treatment of inflammatory and oncologic diseases. Curr. Drug Targets.

[36] Markovic M., Ben-Shabat S., Aponick A., Zimmermann E.M., Dahan A. (2020). Lipids and lipid-processing pathways in drug delivery and therapeutics. Int. J. Mol. Sci..

[37] Markovic M., Ben-Shabat S., Keinan S., Aponick A., Zimmermann E.M., Dahan A. (2018). Prospects and challenges of phospholipid-based prodrugs. Pharmaceutics.

[38] Dahan A., Duvdevani R., Shapiro I., Elmann A., Finkelstein E., Hoffman A. (2008). The oral absorption of phospholipid prodrugs: In Vivo and In Vitro mechanistic investigation of trafficking of a lecithin-valproic acid conjugate following oral administration. J. Control. Release J. Control. Release Soc..

[39] Dahan A., Ben-Shabat S., Cohen N., Keinan S., Kurnikov I., Aponick A., Zimmermann E.M. (2016). Phospholipid-based prodrugs for drug targeting in inflammatory bowel disease: Computational Optimization and In-Vitro Correlation. Curr. Top. Med. Chem..

[40] Dahan A., Markovic M., Keinan S., Kurnikov I., Aponick A., Zimmermann E.M., Ben-Shabat S. (2017). Computational modeling and in-vitro/in-silico correlation of phospholipid-based prodrugs for targeted drug delivery in inflammatory bowel disease. J. Comput. Aided Mol. Des..

[41] Markovic M., Ben-Shabat S., Keinan S., Aponick A., Zimmermann E.M., Dahan A. (2019). Molecular modeling-guided design of phospholipid-based prodrugs. Int. J. Mol. Sci..

[42] Markovic M., Dahan A., Keinan S., Kurnikov I., Aponick A., Zimmermann E.M., Ben-Shabat S. (2019). Phospholipid-based prodrugs for colon-targeted drug delivery: Experimental study and in-silico simulations. Pharmaceutics.

[43] Dahan A., Markovic M., Epstein S., Cohen N., Zimmermann E.M., Aponick A., Ben-Shabat S. (2017). Phospholipid-drug conjugates as a novel oral drug targeting approach for the treatment of inflammatory bowel disease. Eur. J. Pharm. Sci. J. Eur. Fed. Pharm. Sci..

[44] Dahan A., Duvdevani R., Dvir E., Elmann A., Hoffman A. (2007). A novel mechanism for oral controlled release of drugs by continuous degradation of a phospholipid prodrug along the intestine: In-Vivo and In-Vitro evaluation of an indomethacin-lecithin conjugate. J. Control. Release J. Control. Release Soc..

[45] Dvir E., Elman A., Simmons D., Shapiro I., Duvdevani R., Dahan A., Hoffman A., Friedman J.E. (2007). DP-155, a lecithin derivative of indomethacin, is a novel nonsteroidal antiinflammatory drug for analgesia and Alzheimer’s disease therapy. CNS Drug Rev..

[46] Dvir E., Friedman J.E., Lee J.Y., Koh J.Y., Younis F., Raz S., Shapiro I., Hoffman A., Dahan A., Rosenberg G. (2006). A novel phospholipid derivative of indomethacin, DP-155 [mixture of 1-steroyl and 1-palmitoyl-2-{6-[1-(p-chlorobenzoyl)-5-methoxy-2-methyl-3-indolyl acetamido]hexanoyl}-sn-glycero-3-phosophatidyl [corrected] choline], shows superior safety and similar efficacy in reducing brain amyloid beta in an Alzheimer’s disease model. J. Pharmacol. Exp. Ther..

[47] Linderoth L., Fristrup P., Hansen M., Melander F., Madsen R., Andresen T.L., Peters G.H. (2009). Mechanistic study of the sPLA2-mediated hydrolysis of a thio-ester pro anticancer ether lipid. J. Am. Chem. Soc..

[48] Barré A., Azzouz R., Gembus V., Papamicaël C., Levacher V. (2019). Design, synthesis, and In Vitro biological activities of a bio-oxidizable prodrug to deliver both ChEs and DYRK1A inhibitors for AD therapy. Molecules.

[49] Chen K., Aowad A.F., Adelstein S.J., Kassis A.I. (2007). Molecular-docking-guided design, synthesis, and biologic evaluation of radioiodinated quinazolinone prodrugs. J. Med. Chem..

[50] Sun J., Dahan A., Walls Z.F., Lai L., Lee K.D., Amidon G.L. (2010). Specificity of a prodrug-activating enzyme hVACVase: The leaving group effect. Mol. Pharm..

[51] Kim I., Chu X.Y., Kim S., Provoda C.J., Lee K.D., Amidon G.L. (2003). Identification of a human valacyclovirase: Biphenyl hydrolase-like protein as valacyclovir hydrolase. J. Biol. Chem..

[52] Kim I., Crippen G.M., Amidon G.L. (2004). Structure and specificity of a human valacyclovir activating enzyme: A homology model of BPHL. Mol. Pharm..

[53] Lai L., Xu Z., Zhou J., Lee K.-D., Amidon G.L. (2008). Molecular basis of prodrug activation by human valacyclovirase, an alpha-amino acid ester hydrolase. J. Biol. Chem..

[54] Gupta S.V., Gupta D., Sun J., Dahan A., Tsume Y., Hilfinger J., Lee K.D., Amidon G.L. (2011). Enhancing the intestinal membrane permeability of zanamivir: A carrier mediated prodrug approach. Mol. Pharm..

[55] Sun J., Dahan A., Amidon G.L. (2010). Enhancing the intestinal absorption of molecules containing the polar guanidino functionality: a double-targeted prodrug approach. J. Med. Chem..

[56] Karaman R., Dajani K.K., Qtait A., Khamis M. (2012). Prodrugs of acyclovir--a computational approach. Chem. Biol. Drug Des..

[57] Sousa S.F., Ribeiro A.J., Coimbra J.T., Neves R.P., Martins S.A., Moorthy N.S., Fernandes P.A., Ramos M.J. (2013). Protein-ligand docking in the new millennium—A retrospective of 10 years in the field. Curr. Med. Chem..

[58] Kohen A., Cannio R., Bartolucci S., Klinman J.P. (1999). Enzyme dynamics and hydrogen tunnelling in a thermophilic alcohol dehydrogenase. Nature.

[59] Mulholland A.J. (2005). Modelling enzyme reaction mechanisms, specificity and catalysis. Drug Discov. Today.

[60] Ridder L., Harvey J.N., Rietjens I.M.C.M., Vervoort J., Mulholland A.J. (2003). Ab Initio QM/MM modeling of the hydroxylation step in p-Hydroxybenzoate hydroxylase. J. Phys. Chem. B.

[61] Ridder L., Rietjens I.M.C.M., Vervoort J., Mulholland A.J. (2002). Quantum mechanical/molecular mechanical free energy simulations of the glutathione S-Transferase (M1-1) reaction with phenanthrene 9,10-oxide. J. Am. Chem. Soc..

[62] Pinzi L., Rastelli G. (2019). Molecular docking: Shifting paradigms in drug discovery. Int. J. Mol. Sci..

[63] Gao J., Truhlar D.G. (2002). Quantum mechanical methods for enzyme kinetics. Annu. Rev. Phys. Chem..

[64] Kollman P. (1993). Free energy calculations: Applications to chemical and biochemical phenomena. Chem. Rev..

[65] Najjar A., Karaman R. (2019). The prodrug approach in the era of drug design. Expert Opin. Drug Deliv..

[66] Markovic M., Ben-Shabat S., Keinan S., Aponick A., Zimmermann E.M., Dahan A. (2019). Lipidic prodrug approach for improved oral drug delivery and therapy. Med. Res. Rev..

[67] Han H.K., Amidon G.L. (2000). Targeted prodrug design to optimize drug delivery. AAPS Pharmsci.

[68] Pedersen P.J., Adolph S.K., Subramanian A.K., Arouri A., Andresen T.L., Mouritsen O.G., Madsen R., Madsen M.W., Peters G.H., Clausen M.H. (2010). Liposomal formulation of retinoids designed for enzyme triggered release. J. Med. Chem..

[69] Linderoth L., Andresen T.L., Jørgensen K., Madsen R., Peters G.H. (2008). Molecular basis of phospholipase A2 activity toward phospholipids with sn-1 substitutions. Biophys. J..

[70] Karaman R., Hallak H. (2010). Computer-assisted design of pro-drugs for antimalarial atovaquone. Chem. Biol. Drug Des..

[71] Chen K., Wang K., Kirichian A.M., Al Aowad A.F., Iyer L.K., Adelstein S.J., Kassis A.I. (2006). In silico design, synthesis, and biological evaluation of radioiodinated quinazolinone derivatives for alkaline phosphatase-mediated cancer diagnosis and therapy. Mol. Cancer Ther..

[72] Hermann J.C., Hensen C., Ridder L., Mulholland A.J., Holtje H.D. (2005). Mechanisms of antibiotic resistance: QM/MM modeling of the acylation reaction of a class A beta-lactamase with benzylpenicillin. J. Am. Chem. Soc..

